# A nuclear-localized cysteine desulfhydrase plays a role in fruit ripening in tomato

**DOI:** 10.1038/s41438-020-00439-1

**Published:** 2020-12-14

**Authors:** Kang-Di Hu, Xiao-Yue Zhang, Gai-Fang Yao, Yu-Lei Rong, Chen Ding, Jun Tang, Feng Yang, Zhong-Qin Huang, Zi-Mu Xu, Xiao-Yan Chen, Yan-Hong Li, Lan-Ying Hu, Hua Zhang

**Affiliations:** 1 School of Food and Biological Engineering, Hefei University of Technology, 230009 Hefei, China; 2 Xuzhou Institute of Agricultural Sciences of the Xuhuai District of Jiangsu Province, 221131 Xuzhou, China; 3 School of Resources and Environmental Engineering, Hefei University of Technology, 230009 Hefei, China

**Keywords:** Molecular biology, Metabolism, Plant physiology

## Abstract

Hydrogen sulfide (H_2_S) is a gaseous signaling molecule that plays multiple roles in plant development. However, whether endogenous H_2_S plays a role in fruit ripening in tomato is still unknown. In this study, we show that the H_2_S-producing enzyme l-cysteine desulfhydrase SlLCD1 localizes to the nucleus. By constructing mutated forms of SlLCD1, we show that the amino acid residue K24 of SlLCD1 is the key amino acid that determines nuclear localization. Silencing of *SlLCD1* by TRV-SlLCD1 accelerated fruit ripening and reduced H_2_S production compared with the control. A SlLCD1 gene-edited mutant obtained through CRISPR/Cas9 modification displayed a slightly dwarfed phenotype and accelerated fruit ripening. This mutant also showed increased cysteine content and produced less H_2_S, suggesting a role of SlLCD1 in H_2_S generation. Chlorophyll degradation and carotenoid accumulation were enhanced in the *SlLCD1* mutant. Other ripening-related genes that play roles in chlorophyll degradation, carotenoid biosynthesis, cell wall degradation, ethylene biosynthesis, and the ethylene signaling pathway were enhanced at the transcriptional level in the *lcd1* mutant. Total RNA was sequenced from unripe tomato fruit treated with exogenous H_2_S, and transcriptome analysis showed that ripening-related gene expression was suppressed. Based on the results for a *SlLCD1* gene-edited mutant and exogenous H_2_S application, we propose that the nuclear-localized cysteine desulfhydrase SlLCD1 is required for endogenous H_2_S generation and participates in the regulation of tomato fruit ripening.

## Introduction

Hydrogen sulfide (H_2_S) is a gaseous signaling molecule that is widely present in living organisms. Accumulating evidence has confirmed multiple functions of H_2_S in plant root development, stomatal movement, postharvest senescence, petiole abscission, and response to abiotic stresses^1–6^. Similar to the role of nitric oxide in the regulation of fruit ripening and senescence, multiple studies have found that H_2_S can alleviate postharvest ripening and senescence of fruits, such as strawberry, kiwifruit, and banana, by regulating the antioxidant system and ethylene pathway^2,7,8^. H_2_S fumigation could reduce the fruit decay index and respiratory intensity of strawberry^2^. Exogenous H_2_S could act as a regulator of fruit ripening by antagonizing the effect of ethylene in tomatoes^9^. Transcriptome analysis indicated that H_2_S could delay the ripening and senescence of kiwifruit by modulating genes involved in cell wall degradation and in the ethylene signaling pathway^10^. H_2_S could also perform a signaling role through persulfidation, which is the posttranslational modification of cysteine residues (R-SHs) in target proteins by covalent addition of thiol groups to form persulfides (R-SSHs)^11,12^. For instance, H_2_S was found to inhibit ethylene synthesis by inhibiting the activity of 1-aminocyclopropane-1-carboxylic acid (ACC) oxidases (ACOs) by persulfidation, thereby alleviating tomato ripening and senescence^12^. Previous research on the role of H_2_S in alleviating fruit ripening and senescence has been conducted mainly through exogenous H_2_S fumigation, while whether endogenous H_2_S generation affects fruit ripening is still unclear.

In plants, endogenous H_2_S can be produced through the sulfate assimilation pathway in chloroplasts, where the enzyme is sulfite reductase (SiR)^13^. Endogenous cytosolic H_2_S could also be generated from l-cysteine by cysteine-degrading enzymes^14^. l-cysteine desulfhydrase 1 (DES1), belonging to the O-acetylserine thiol lyase (OASTL) family, was characterized as the key enzyme in this process^15^. LCD (l-cysteine desulfhydrase, with l-Cys as the substrate) has also been shown to catalyze the degradation of cysteine to H_2_S, ammonia, and pyruvate. In Arabidopsis, the H_2_S-deficient mutant *lcd* displayed increased stomatal density and stomatal index values, and H_2_S was found to act downstream of jasmonic acid signaling to regulate stomatal development in cotyledons^16^. Furthermore, *LCD* expression was activated by the Ca^2+^/calmodulin2-binding transcription factor TGA3 in Arabidopsis to increase H_2_S production and bolster Cr^6+^ tolerance^3^.

Ethylene is a key hormone in the regulation of fruit ripening and senescence, especially in respiratory climacteric fruits such as tomato^17^. The ethylene synthesis pathway has been characterized in tomato, and increased expression of *ACS1A*, *ACS2*, *ACS4*, *ACO1*, and *ACO3*, genes encoding ACC synthase and ACC oxidase, play an important role in tomato fruit ripening^18^. The transition from green to red is one of the phenotypic characteristics of fruit ripening in tomato. Chlorophyll b reductase (NYC1), pheophytinase (PPH), pheophorbide a oxygenase (PAO), and SGR1 (stay-green protein) are required for chlorophyll degradation^19^. Carotenoid biosynthesis starts with the condensation of two geranylgeranyl diphosphate (GGPP) molecules by phytoene synthase (PSY) to form phytoene. Phytoene desaturase (PDS) and ζ-carotene desaturase (ZDS) are enzymes that catalyze two symmetric dehydrogenation reactions, converting 15-cis phytoene to tetra-cis-lycopene^17^. In tomato, *PSY1*, *PDS*, and *ZDS* are key genes in the regulation of carotenoid synthesis. During fruit softening, cell wall components undergo degradation by pectin methylesterase, polygalacturonase (PG), cellulase (CEL), and xyloglucan-degrading enzymes (XTHs)^17^.

Our previous research showed that exogenous H_2_S delayed the ripening of postharvest tomato fruits by modulating the antioxidant system and ethylene signaling pathway^9^. In the present research, transcriptome sequencing was performed in tomato fruit treated with H_2_S, and the genes involved in amino acid metabolism and ripening-related genes were shown to be enriched. We found two LCD-encoding genes, namely, *SlLCD1* and *SlLCD2*, in tomato, whereas their role in regulating fruit ripening is unknown. In the present research, the subcellular localization of SlLCD1 was studied in tobacco cells, and the role of SlLCD1 in sulfur metabolism and fruit ripening was studied by virus-induced gene silencing (VIGS) and CRIPSR/Cas9-mediated gene editing in tomato fruit to understand the role of H_2_S in fruit ripening.

## Results

### Expression profile of the *SlLCD* genes in tomato plants

**Fig. 1:**
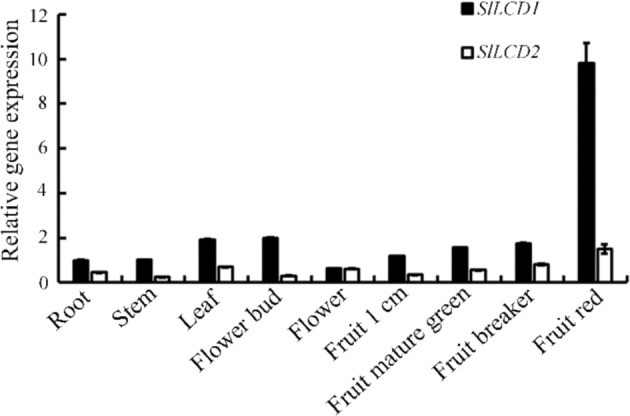
*SlLCD* expression pattern in different tissues of tomato. The tissues include root, stem, leaf, flower bud, flower, 1 cm fruit, mature green fruit, breaker fruit, and red fruit. Values are the means ± SDs of three replicates

The expression profiles of the *SlLCD* genes in tomato plants were analyzed by reverse transcription quantitative polymerase chain reaction (RT-qPCR) (Fig. 1). *SlLCD1* gene expression increased gradually during fruit development and ripening, whereas that of *SlLCD2* did not change too much. The expression of *SlLCDs* was also explored in different tissues of Micro-Tom by analysis of the public database TomExpress http://tomexpress.toulouse.inra.fr/login. The data showed that increased *SlLCD1* expression was observed during fruit ripening, suggesting the possible role of *SlLCD1* and H_2_S in the regulation of fruit ripening (Fig. S1).

### Subcellular localization of SlLCD1 and SlLCD2

**Fig. 2:**
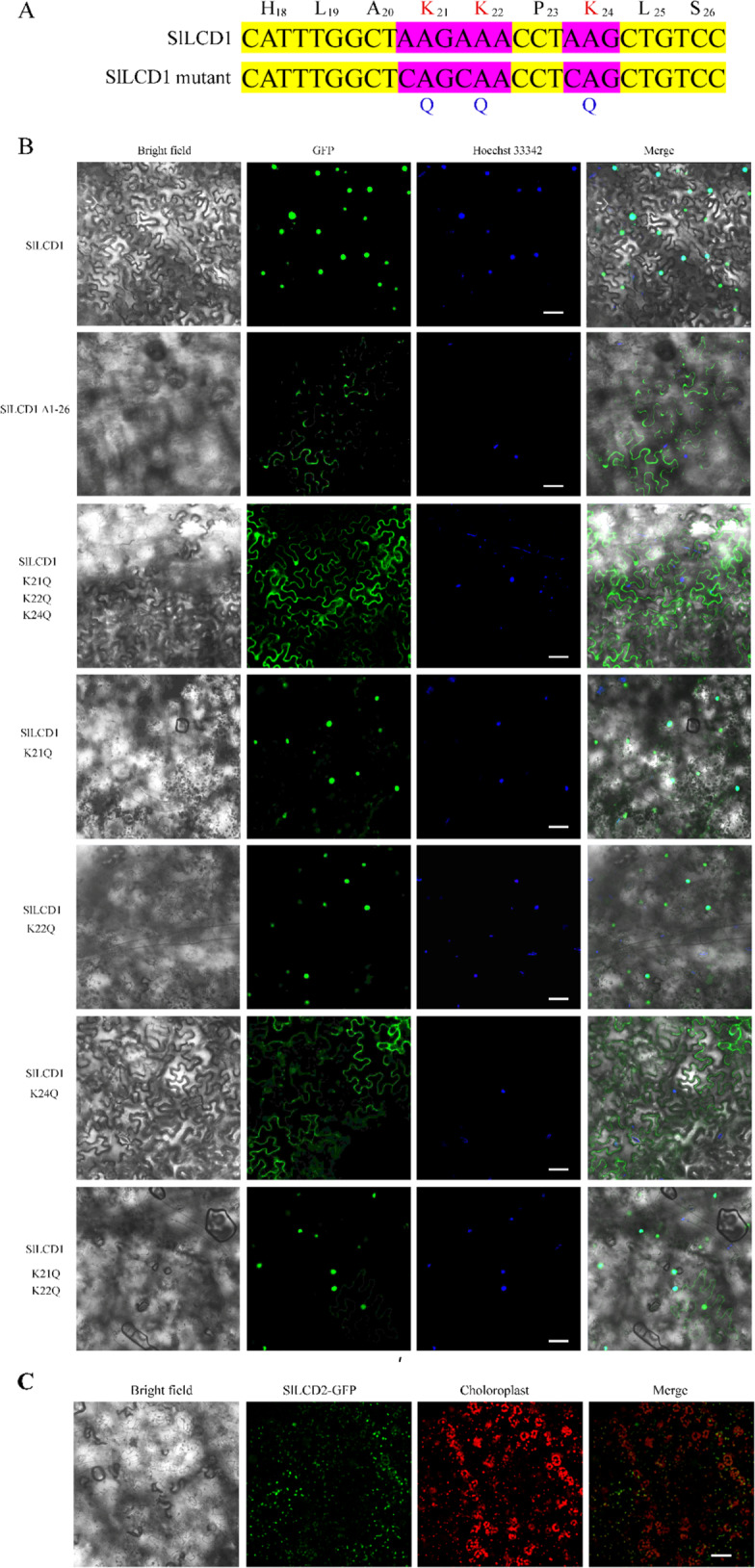
Subcellular localization of the SlLCD1 protein in tobacco cells. **A** The K (lysine) residues in SlLCD1 were substituted with Q (glutamine). **B** Confocal image of tobacco leaf cells expressing SlLCD1-GFP or a truncation (Δ1–26) or the following site-directed mutant GFP fusion proteins: SlLCD1 (K21Q K22Q K24Q)-GFP, SlLCD1 (K21Q)-GFP, SlLCD1 (K22Q)-GFP, SlLCD1 (K24Q)-GFP, and SlLCD1 (K21Q K22Q)-GFP. Hoechst 33342 represents the nucleus. The red fluorescence signal in **C** (SlLCD2-GFP) represents the autofluorescence of chloroplasts. Scale bars represent 50 μm

As SlLCD1 showed a potential nuclear localization signal (NLS) at its N-terminus (H_18_LAKKPKLS), we predicted that SlLCD1-GFP might localize to the nucleus. Confocal and fluorescence microscopy confirmed that SlLCD1 showed strong nuclear localization, as fluorescence from SlLCD1-GFP merged with the nuclear stain Hoechst 33342 in transfected tobacco leaf cells (Fig. 2). Different mutants of SlLCD1 were constructed, and a schematic diagram of the intended mutations in SlLCD1 is shown in Figs. S2 and 2A. To verify whether the NLS segment was essential for the nuclear localization of SlLCD1, a truncation mutant, SlLCD1 (Δ1–26)-GFP, which lacked the first 26 amino acid residues at the N-terminus was constructed, and the truncation mutant failed to localize to the nucleus but was located in the cytoplasm. The basic amino acids lysine (K) and arginine (R) are key factors defining monopartite NLSs; thus, we mutated the native NLS of SlLCD1 to a series of variants by replacing lysine residues with glutamines: SlLCD1 (K21Q K22Q K24Q), SlLCD1 (K21Q), SlLCD1 (K22Q), SlLCD1 (K24Q), and SlLCD1 (K21Q K22Q). Nuclear localization of SlLCD1 was abolished in the SlLCD1 (K21Q K22Q K24Q) mutant, whereas SlLCD1 (K21Q), SlLCD1 (K22Q), and SlLCD1 (K21Q K22Q) retained nuclear localization. Surprisingly, the SlLCD1 (K24Q) mutant failed to localize to the nucleus (Fig. 2B), suggesting that K_24_ in SlLCD1 was essential for the nuclear localization of SlLCD1. As shown in Fig. 2C, SlLCD2-GFP showed strong colocalization with chloroplast autofluorescence, suggesting that SlLCD2 was mainly localized to the chloroplast. The homologous proteins in 16 other plant species were analyzed to determine whether the potential NLS found in SlLCD1 is conserved in other plant species. As shown in Supplementary Table 1, at least one member of the LCD proteins in the plant contained a potential NLS at the N-terminus.

### VIGS of *SlLCD1* accelerates tomato fruit ripening

**Fig. 3:**
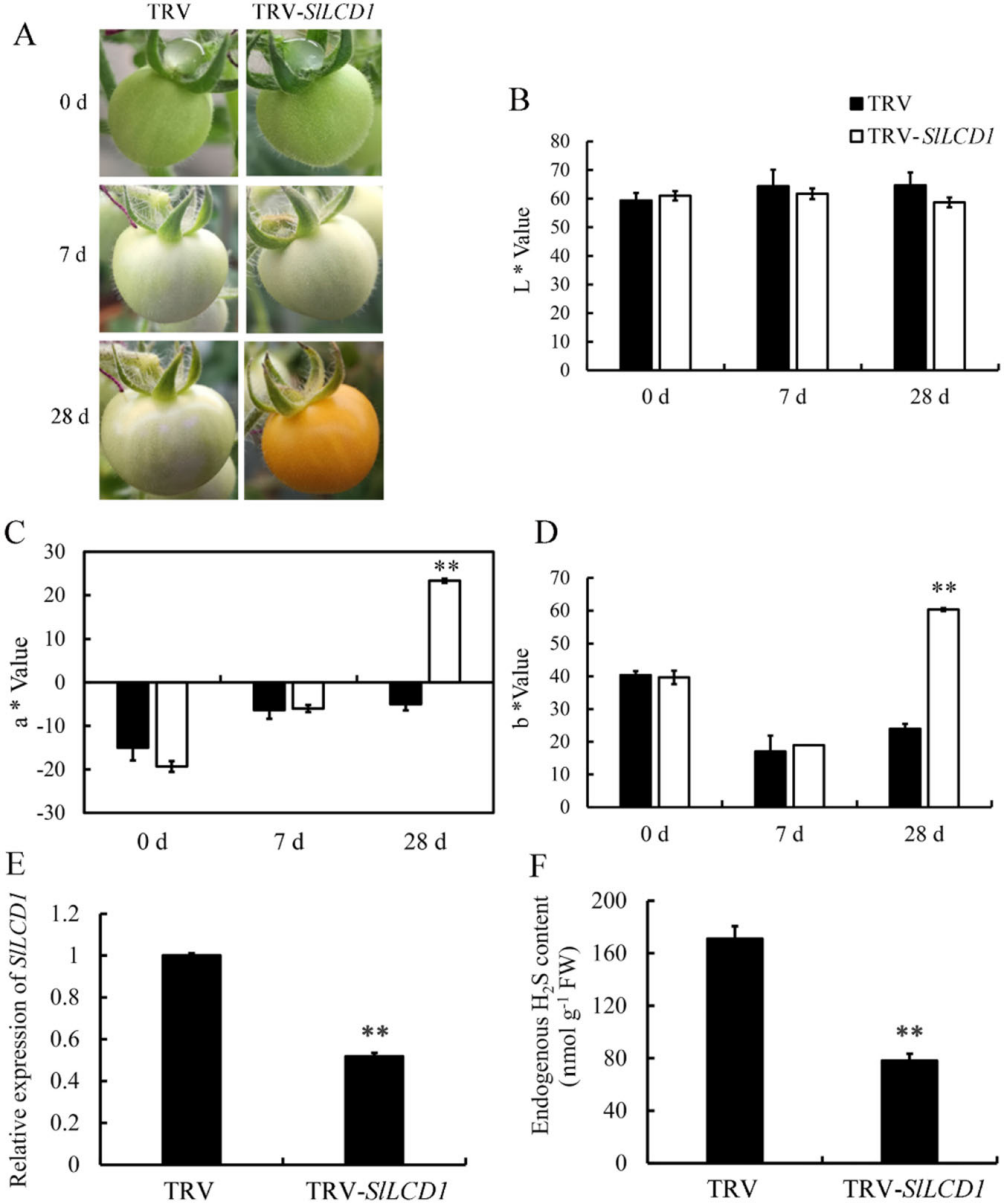
VIGS of *SlLCD1* accelerates tomato fruit ripening. **A** Phenotype of *SlLCD1*-silenced fruits of the Micro-Tom cultivar. Left: tobacco rattle virus (TRV)-infected fruit in which ripening was not affected by TRV. Right: TRV-SlLCD1-infected fruit showed accelerated color transition from green to red. Photos were taken at 0, 7, and 28 days after infection with *Agrobacterium tumefaciens* strain GV3101 containing the vectors. **B** L* indicates lightness. **C** The a* value stands for chromaticity on a green (−) to red (+) axis. **D** b* Indicates chromaticity on the blue (−) to yellow (+) axis. Values are the means ± SDs of three replicates. The symbol ** stands for *P* < 0.01. The gene expression of *SlLCD1* (**E**) and the content of H_2_S (**F**) in *SlLCD1*-silenced fruits (silenced by VIGS). Values are the means ± SDs of three replicates. The symbol ** stands for *P* < 0.01

VIGS constructs containing the tobacco rattle virus (TRV)-LCD1 were used to silence the expression of *LCD1*. As shown in Fig. 3A, fruit infected with TRV-LCD1 showed pink coloration on day 28 after infection, while control fruit infected with the empty vector remained white-green at day 28. Chromaticity data showed that *LCD1*-silenced fruit exhibited a higher a value on the green (−) to red (+) axis compared with control fruit and a higher b* value on the blue (−) to yellow (+) axis (Fig. 3C, D), whereas the value of lightness (*L*) was not significantly changed (Fig. 3B). The silencing effect of VIGS-LCD1 was confirmed by RT-qPCR, as shown in Fig. 3E. The expression of *LCD1* was downregulated significantly by VIGS and reached approximately half of that in control fruit. LCD catalyzes the generation of H_2_S with cysteine as its substrate; thus, in fruit where *LCD1* is silenced, a decrease in the H_2_S content in fruit might be expected. Accordingly, Fig. 3F shows that tomato fruit harboring the VIGS-LCD1 construct contained a significantly lower level of H_2_S than the control.

### Generation of the *LCD1* mutation by CRISPR/Cas9

**Fig. 4:**
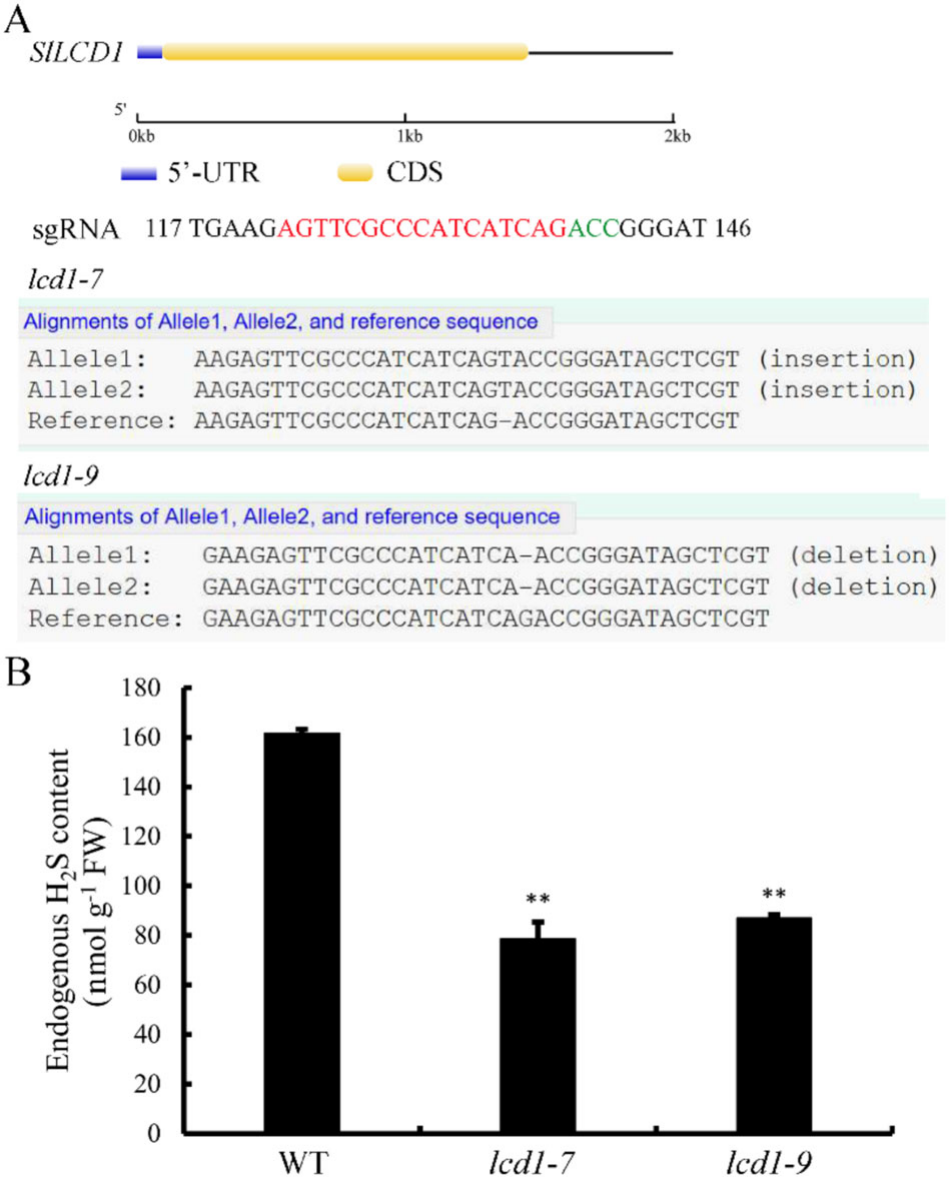
Identification of *SlLCD1* gene-edited plants. **A** Gene structure of *SlLCD1*, which is indicated as the 5’-UTR (untranslated region) and CDS (coding sequence). Generation of *SlLCD1* mutations by CRISPR/Cas9 using single-guide RNAs (sgRNAs). Sequences of the *SlLCD1* mutants *lcd1-7* and *lcd1-9* are shown. sgRNA target and a protospacer-adjacent motif (PAM) are indicated in red and green, respectively. Deletions and insertions are indicated by dashes. **B** Endogenous H_2_S content in leaves of the wild type (WT) and the *lcd1-7* and *lcd1-9* mutants of tomato. The values are the means ± SDs of three replicates. The symbol ** stands for *P* < 0.01

For stable transformation of *SlLCD1*, the CRISPR/Cas9 vector containing two sgRNA targets of *LCD1* was generated and introduced into *Agrobacterium tumefaciens* strain GV3101 for tomato transformation. For the positive T1 plants, the gene fragment of *SlLCD1* was amplified with genomic DNA as a template with primers that flanked both sgRNA targets. As shown in Fig. 4A, sequencing showed that *lcd1-7* contained a T residue inserted near the PAM sequence, and *lcd1-9* had a deletion of G near the PAM. The sequence surrounding the first sgRNA is shown, while the second sgRNA is not shown, as the nearby sequence was not edited. Both the insertion and deletion of a single base led to the formation of a premature stop codon, eliminating the cysteine desulfhydrase domain of LCD1. Specifically, the T insertion in *lcd1-7* caused a severe frameshift mutation, and the translation stopped after the 53rd amino acid residue. In *lcd1-9*, the one-base deletion caused a mutated peptide sequence after the 46th amino acid residue, and the translation stopped at the 48th amino acid due to a premature stop codon. To confirm that this mutation affected *LCD1* function, we measured the endogenous H_2_S content of wild-type and *lcd1-7*/*9* mutant plants. Figure 4B shows that mutation of both *lcd1-7* and *lcd1-9* resulted in significantly lower levels of H_2_S in comparison to the wild type, suggesting that the mutations altered the enzymatic function of LCD1 as a cysteine desulfhydrase. The enzymatic activity of SlLCDs was also confirmed by ectopic expression in *Escherichia coli*, and the result in Fig. S3 shows that both SlLCDs could catalyze the production of H_2_S with l-cysteine as the substrate.

### Role of LCD1 in tomato fruit ripening

**Fig. 5:**
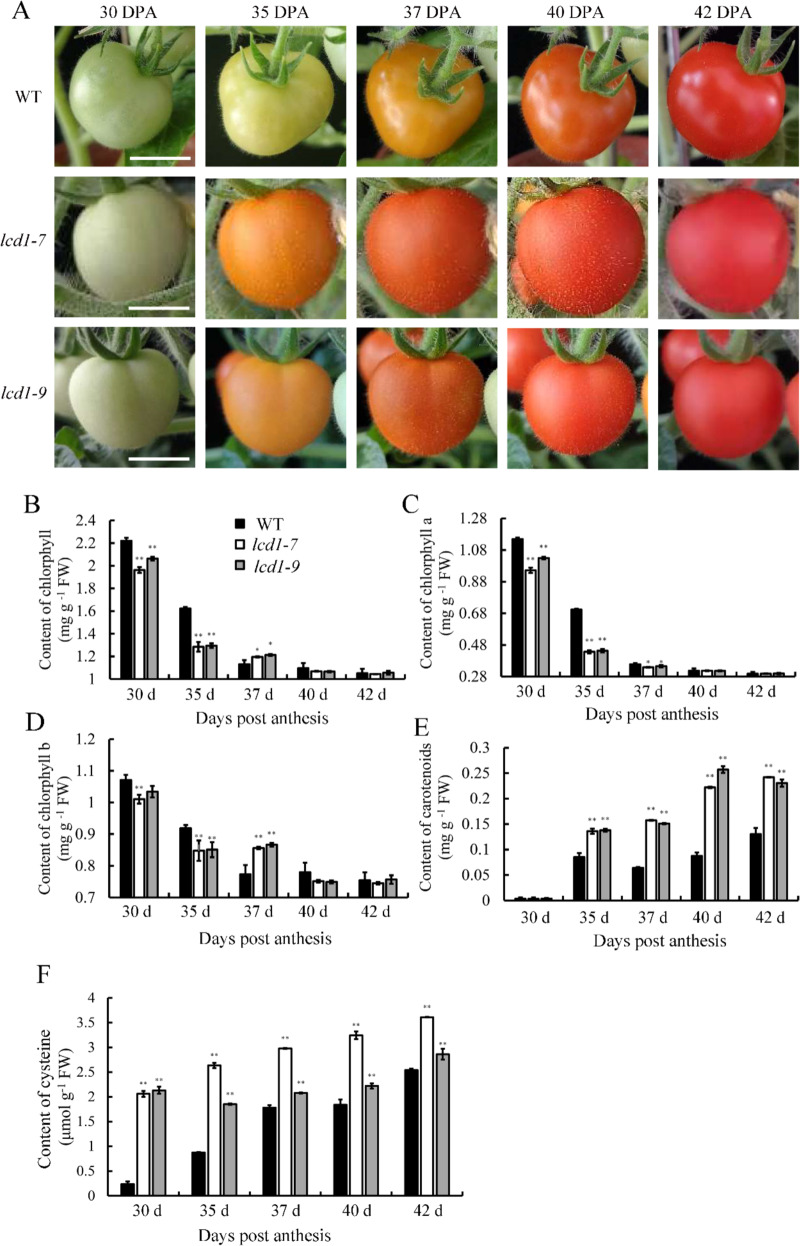
*SlLCD1* mutation caused accelerated fruit ripening in tomato. **A** Fruit phenotypes of *lcd1-7* and *lcd1-9* mutant plants during 42 days post anthesis (DPA). **B**–**F** The levels of total chlorophyll, chlorophyll a, chlorophyll b, carotenoids, and cysteine in fruit of wild-type and mutant plants during 42 days post anthesis (DPA). The values are the means ± SDs of three replicates. The symbol ** stands for *P* < 0.01. Bar 1 cm

We characterized the phenotypes of the WT and *lcd1* mutant tomato plants by measuring plant heights and fruit coloration. As shown in Fig. S4, both *lcd1-7* and *lcd1-9* led to shorter plant heights, suggesting that mutation of *LCD1* affected the growth of tomato plants. The role of the *LCD1* mutation in fruit ripening was observed in the first flowering branch. As shown in Fig. 5A, tomato fruit turned pink at 37 days post anthesis (DPA) and became fully red at 40 DPA. In contrast, the *lcd1-7* and *lcd1-9* mutants turned orange at 35 DPA, suggesting that *LCD1* played a negative role in fruit ripening. We also measured tomato fruit ripening by determining the levels of chlorophylls and carotenoids. As shown in Fig. 5B, the total chlorophyll content decreased gradually from 30 to 42 DPA in the wild-type and *lcd1* mutants. However, the *LCD1* mutation caused significantly lower chlorophyll content at 30 and 35 DPA compared with the wild type, and similar trends were observed when chlorophylls a and b were measured (Fig. 5C, D). Figure 5E shows changes in carotenoids during fruit ripening in the wild type and *lcd1* mutants. The carotenoid content increased gradually from 30 to 42 DPA in the wild type, while much higher levels were observed in the *lcd1* mutants. At 37 DPA and 42 DPA, the carotenoid content in the *lcd1* mutants was approximately two times that in wild-type fruit. Overall, the metabolism of pigments further suggested the role of *LCD1* in the regulation of fruit ripening.

Due to the role of LCD in catalyzing cysteine synthesis^3,14^, we speculated that cysteine might accumulate in the *lcd1* mutant, which was confirmed by the data shown in Fig. 5F. Cysteine levels accumulated gradually in the wild type during fruit ripening. However, significantly higher amounts of cysteine were observed in *lcd1-7*/*9* at 30 DPA, where the cysteine content in *lcd1* mutant fruit was approximately ten times that in wild-type fruit. At 35–42 DPA, the cysteine content of *lcd1-9* was lower than that of *lcd1-7* but still significantly higher than that of the wild type. The increased cysteine content in *lcd1* mutant fruit suggested that the *LCD1* mutation failed to decompose cysteine, which resulted in accumulated cysteine.

### Effects of the *LCD1* mutation on the expression of ripening-related genes

**Fig. 6:**
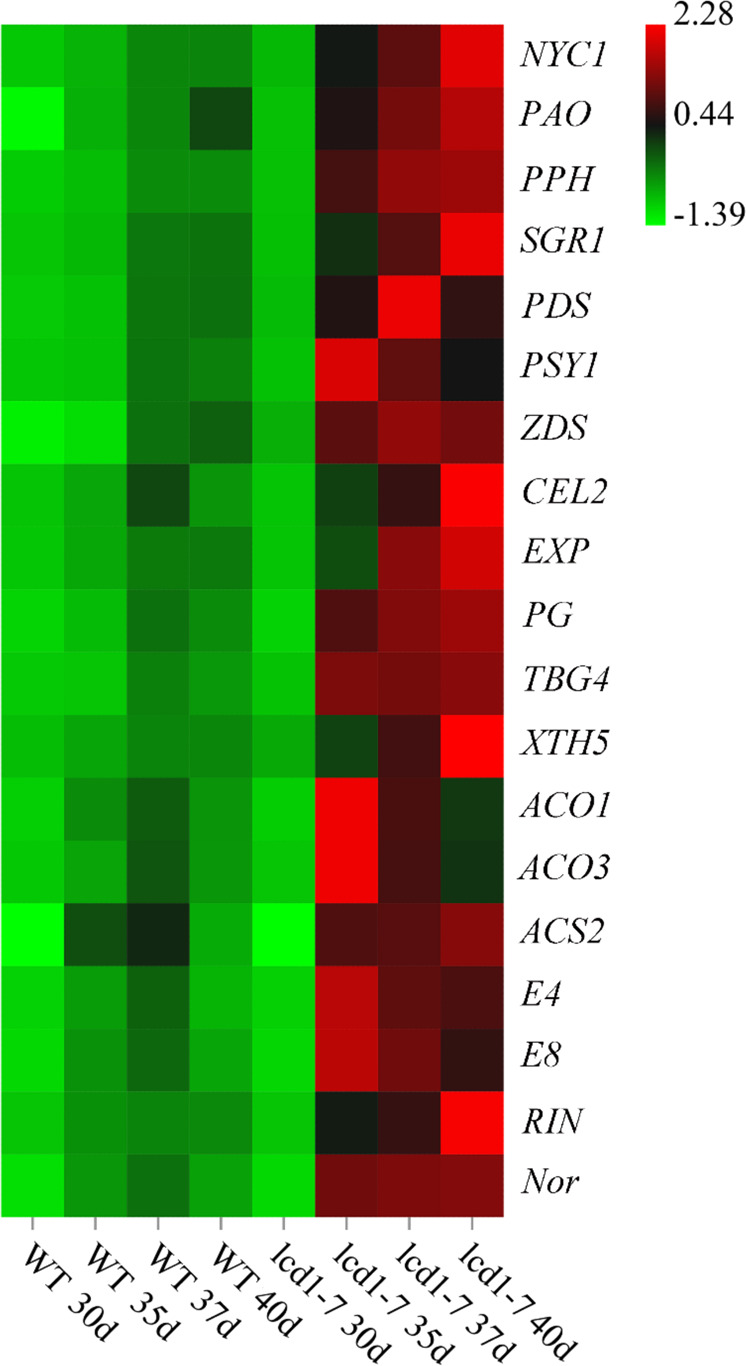
*SlLCD1* mutation increases the expressions of ripening related genes during tomato fruit ripening. Heatmap of the relative gene expression of *NYC1*, *PAO*, *PPH*, *SGR1*, *PDS*, *PSY1*, *ZDS*, *CEL2*, *EXP*, *PG*, *TBG4*, *XTH5 ACO1*, *ACO3*, *ACS2*, *E4*, *E8*, *RIN*, and *Nor* in fruit of wild-type and *lcd1-7* mutant plants at 40 days post anthesis (DPA)

To explore the molecular mechanism underlying the difference in chlorophyll content during the ripening process of wild-type and *lcd1-7* tomato, the relative expression levels of the key genes involved in the chlorophyll degradation pathway, i.e., *NYC1*, *PAO*, *PPH*, and *SGR1*, were analyzed. As shown in the heatmap (Fig. 6) and bar chart in Fig. S5, the expression levels of the *NYC1*, *PAO*, *PPH*, and *SGR1* genes were significantly increased in the fruit with the *LCD1* mutation during ripening. Although increased gene expression of *NYC1*, *PAO*, *PPH*, and *SGR1* was observed in the wild type, the levels were lower than those in *lcd1* mutant fruit. Among these genes, the expression levels of the *PPH* and *SGR1* genes in the fruit of the *lcd1* mutant at 35 DPA were nearly ten times that in wild-type fruit. Thus, it could be concluded that increased gene expression in the chlorophyll degradation pathway may contribute to accelerated chlorophyll degradation in *lcd1* mutant fruit.

Because the *LCD1* mutation caused carotenoid accumulation in fruit, the expression of *PSY1*, *PDS*, and *ZDS* involved in carotenoid biosynthesis was analyzed in the wild type and in the *lcd1* mutant. Figure 6 shows that the expression of the *PDS*, *PSY1*, and *ZDS* genes was significantly increased in *lcd1* mutant fruit compared with that in the wild type. For example, the expression of the *PSY1* gene in fruit of the *lcd1* mutant was 74 times that in fruit of the wild type at 35 DPA (Fig. S5).

To establish a role for *SlLCD1* in the metabolism of cell wall components in tomato fruit, the expression levels of genes encoding enzymes that play a role in cell wall biosynthesis and degradation were measured in wild-type and *lcd1* mutant fruit. Figures 6 and S6 show that the expression of *CEL2* (endoglucanase), *EXP* (expansin), *XTH5* (xyloglucan endotransglucosylase), *PG* (polygalacturonase), and *TBG4* (beta-galactosidase) was upregulated in *lcd1* tomato fruit compared to wild-type fruit, though the gene expression levels increased gradually during fruit ripening in both the wild type and *lcd1-7*. Expression of *CEL2* in *lcd1-7* mutant fruit was upregulated approximately 9-fold at 40 DPA compared to its expression in the wild type. *PG* and *TBG4* were upregulated 10-fold and 47-fold at 35 DPA in *lcd1-7* mutant fruit relative to the wild type (Figs. 6 and S6).

Tomato releases ethylene during fruit ripening by upregulating the expression of genes of the ethylene biosynthesis pathway; therefore, we analyzed these genes in wild-type and *lcd1-7* tomato fruit. Figure 6 shows the expression of the ethylene biosynthesis genes *ACO1*, *ACO3*, and *ACS2*. Their expression increased gradually in wild-type fruit, suggesting that they were the marker genes of fruit ripening, but the expression levels of *ACO1*, *ACO3*, and *ACS2* in *lcd1-7* were higher than those in wild-type fruit. Among these genes, the expression of *ACO1* and *ACO3* in *lcd1-7* was upregulated by 6-fold and 10-fold, respectively, at 35 DPA compared with the expression in the wild type (Fig. S7).

*E4* (encoding the peptide methionine sulfoxide reductase msrA) and *E8* (1-aminocyclopropane-1-carboxylate oxidase-like protein) are important ethylene-responsive marker genes in tomato^16^. As shown in Fig. 6, the relative expression levels of *E4* and *E8* were upregulated in tomato fruit. The *lcd1* mutant showed upregulation of *E4* by approximately 7-fold and 8-fold at 35 and 40 DPA, respectively, compared with the wild type (Fig. S7). *RIN*, a MADS-box family transcription factor, is a positive regulator of tomato fruit ripening and plays a role in the ethylene synthesis pathway. Nor, an NAC family transcription factor, also functions upstream of ethylene synthesis to control fruit ripening. Figure 6 shows that the expression levels of *RIN* and *Nor* increased at the beginning of fruit ripening; however, the *lcd1* mutation led to increased gene expression. *RIN* and *Nor* gene expression in *lcd1* tomato fruit was approximately 5- and 6.7-fold that in the wild type at 40 DPA (Fig. S7).

### Exogenous H_2_S treatment delayed tomato fruit ripening and attenuated the expression of ripening-related genes

**Fig. 7:**
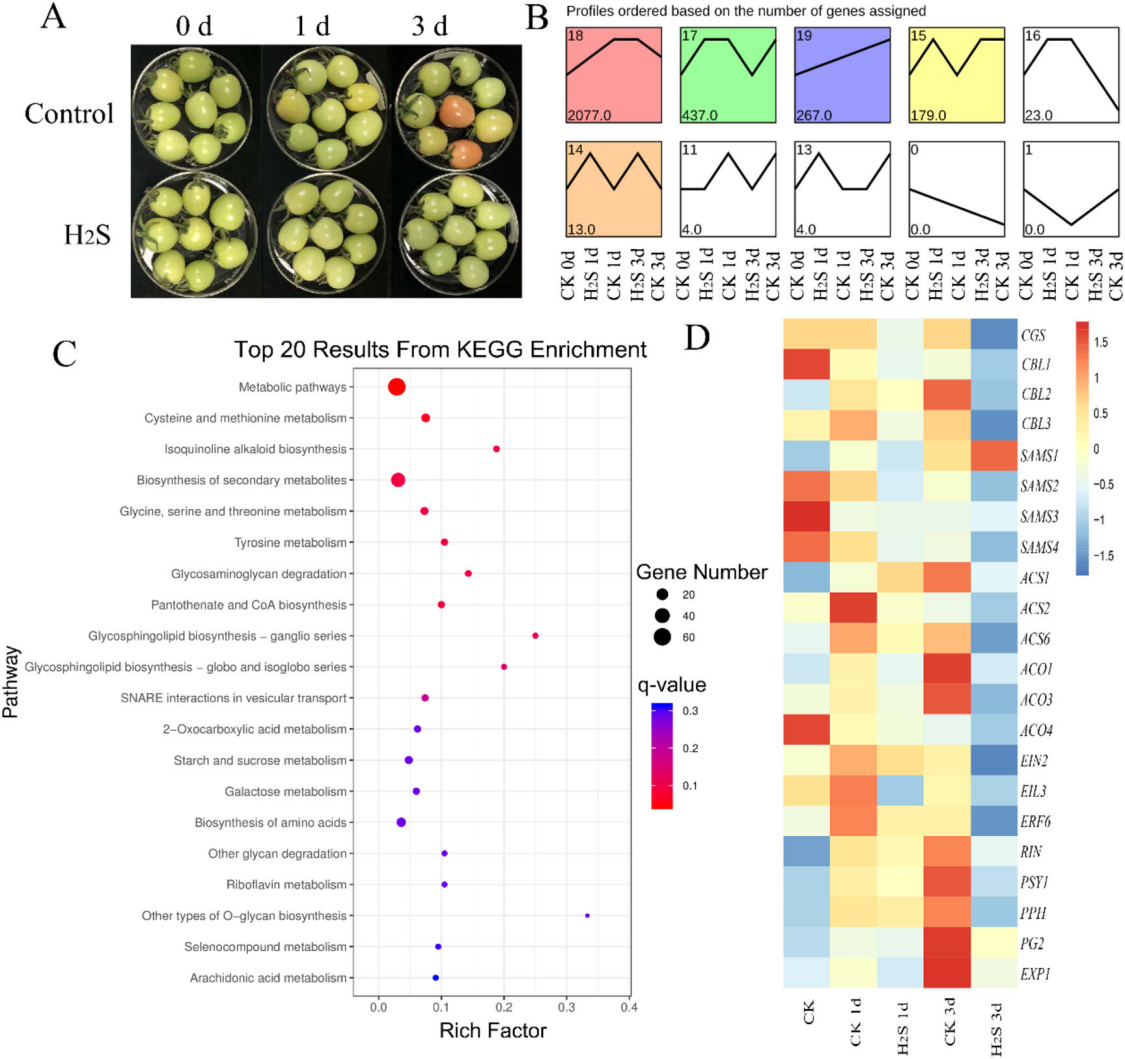
Transcriptomic analysis of tomato fruit treated with H_2_S (H_2_S) or the control (CK) for 0, 1, and 3 days. **A** Images of tomato fruit with H_2_S (H_2_S) or the control (CK) for 0, 1, and 3 days. **B** Differential gene expression pattern analysis based on transcriptomic data. **C** KEGG analysis based on the genes in profile 17 as shown in **B**. **D** Heatmap of gene expression data of ripening-related genes in tomato fruit treated with H_2_S (H_2_S) or the control (CK) for 0, 1, and 3 days

Tomato fruits at the white mature stage that were fumigated with H_2_S showed markedly delayed fruit ripening (Fig. 7A). To explore the role of H_2_S in this process, fruit were sampled for transcriptomic analysis at 0, 1, and 3 days post storage, and the expression of ripening-related genes was analyzed. The data in Fig. S8A show that H_2_S treatment for 1 day induced the expression of 4188 genes and reduced the expression of 4911 genes in comparison to control fruit. On day 3, 9988 genes were upregulated and 6232 genes were downregulated in H_2_S-treated fruit compared with the control, suggesting that H_2_S induced profound changes in gene expression. Gene expression patterns were analyzed by using the Kyoto Encyclopedia of Genes and Genomes (KEGG) database as shown in Fig. 7B. Profile 17 was enriched in genes that showed upregulation on day 3 for control fruit. Figures 7C and S8B–E show that the genes in profile 17 are involved in the cysteine and methionine metabolism pathways, selenocompound metabolism, porphyrin and chlorophyll metabolism, and plant hormone signal transduction. Then, the genes that showed upregulation upon H_2_S treatment in profiles 14 and 15 were also analyzed, and the results in Fig. S9 show that H_2_S treatment affects many metabolic pathways, including primary and secondary metabolism. In addition, the differentially expressed genes in H_2_S-treated fruit compared with the control on days 1 and 3 were analyzed through KEGG pathway analysis (Figs. S10–13). Generally, H_2_S treatment upregulated the pathways of ribosome biogenesis, plant hormone signal transduction, glutathione metabolism, ABC transporters, and plant–pathogen interaction compared with the control. In contrast, the pathway of carotenoid biosynthesis was inhibited by H_2_S.

We also asked whether the ripening-related genes in tomato fruit were modulated by H_2_S. The heatmap in Fig. 7D shows that many genes in the methionine pathway, including *CGS* (cystathionine gamma synthase), *CBL1* (cystathionine beta-lyase), *CBL2*, *CBL3*, *SAM2* (S-adenosylmethionine synthase), *SAM3*, and *SAM4*, were repressed in H_2_S-treated tomato fruit. The ethylene biosynthesis genes *ACS1*, *ACS2*, *ACS6*, *ACO1*, *ACO3*, and *ACO4* showed increased expression during postharvest storage of tomato, whereas the increase was greatly attenuated by H_2_S treatment. Many genes involved in ethylene signaling, including *EIN2*, *EIL3*, *ERF6*, and *RIN*, were upregulated in control fruit at day 3, and H_2_S inhibited expression. *PSY1*, *PPH*, *PG2*, and *EXP1* (encoding an expansin precursor) are ripening-related genes for chlorophyll degradation and fruit softening, and their expression is also repressed by H_2_S.

## Discussion

The research presented in this paper shows that the cysteine desulfhydrase SlLCD1, a key enzyme in sulfur metabolism in plants, that we identified in tomato is localized to the nucleus, and this localization is mediated by a potential NLS. It had previously been proposed that the cysteine desulfhydrase of *Arabidopsis* (L-CDesI, At3g62130) is a nuclear localized enzyme^14,20^, but this was not supported by direct experimental evidence. Our work provides evidence that amino acid residues at the N-terminus of SILCD1 do indeed encode a functional NLS. We show that the H_18_LAKKPKLS domain of SlLCD1 located at the N-terminus is essential for nuclear localization and that K_24_ is the key amino acid. Mutation of the other lysine residues, namely, K21Q and K22Q, in the putative NLS domain abolished the nuclear localization, and only those sequences containing K24 were located in the nucleus. The detailed mechanism underlying SlLCD1’s nuclear localization is still unknown, but it is likely that importins may mediate its subcellular localization. A major process for the active import of proteins into the nucleus is initiated by the binding of cargo proteins containing NLSs to α‐importins in the cytosol^21^. For instance, Arabidopsis phosphatidylinositol 4-phosphate 5-kinase 2 was reported to contain a functional nuclear localization sequence and interacts with alpha-importins, which mediate its nuclear localization^22^. As SlLCD1 is located in the nucleus, we propose that its substrate cysteine is also present in the nucleus. Recently, it was reported that the O-acetylserine(thiol)lyases SlOAS2, 4, and 6 localized in the membrane, cytosol, and nucleus, suggesting that cysteine could be synthesized in the nucleus^20^. Sulfur-related metabolism is dynamic in the nucleus. For instance, methionine (Met) adenosyltransferase 4 (MAT4)/S-adenosyl-Met synthetase 3, which catalyzes the synthesis of S-adenosyl-Met (SAM), is located in the nucleus in the one-carbon metabolism cycle^23^.

To understand the role of cysteine desulfhydrases in regulating tomato fruit ripening, we used the BLAST tool in the NCBI database using Arabidopsis LCD as the query and identified two LCD genes in tomato. These tomato LCD sequences were used to examine expression patterns in different tissues at different developmental stages using the TomExpress database^24^. Among the two *LCD* genes, *SlLCD1* showed increased gene expression during fruit ripening, which was confirmed by RT-qPCR of *SlLCD1*. When expression of *SlLCD1* was silenced with a VIGS construct containing the TRV, accelerated fruit ripening was observed in VIGS-SlLCD1 compared with plants infected with an empty construct. We confirmed the silencing of VIGS-LCD by RT-qPCR, where the expression of *LCD1* in silenced fruit was half that in the control fruit, and H_2_S production was also reduced in silenced plants (Fig. 3E). These observations provide a clear link between reduced H_2_S content and fruit ripening in tomato when *LCD1* is silenced.

These observations linking reduced H_2_S levels to fruit ripening were confirmed using CRISPR/Cas9 to generate *lcd1* mutants. Both *lcd1-7* and *lcd1-9* showed decreased endogenous H_2_S content and enhanced fruit ripening compared to control tomato plants. We also analyzed chlorophyll and carotenoid levels in the fruit of CRISPR/Cas9 mutants and showed that the *lcd1* mutation resulted in accelerated chlorophyll degradation and enhanced carotenoid accumulation. Surprisingly, we observed increasing cysteine content in both wild-type and *lcd1* mutant fruit. We speculate that elevated cysteine levels may increase methionine levels. Methionine is a key intermediate in ethylene synthesis; thus, accelerated fruit ripening in *lcd1* could be partially attributed to enhanced C_2_H_4_ production via methionine from cysteine^25^.

The expression of ripening-related genes was analyzed in the *lcd1* mutant to study the effect of reduced H_2_S. Consistent with the observation of accelerated chlorophyll degradation in *lcd1*, the expression of *NYC1*, *PAO*, *PPH*, and *SGR1* was significantly enhanced during fruit ripening. Moreover, the expression of *PSY1*, *PDS*, and *ZDS*, genes involved in carotenoid biosynthesis, was also increased in *lcd1* mutant fruit. The modulation of pigment metabolism-related genes may contribute to the degradation of chlorophyll and accumulation of carotenoids in *lcd1* mutant fruit.

Tomato fruit ripening has been shown to be accompanied by changes in the expression of genes that play roles in ethylene synthesis and cell wall modification. Our data show that the cell wall metabolism-related enzymes *CEL2*, *EXP*, *XTH5*, *PG*, and *TBG4*, the ethylene biosynthesis-related genes *ACO1*, *ACO3*, and *ACS2*, and the ethylene responsive genes E4 and E8 were all expressed at significantly higher levels in *lcd1-7* than in wild-type tomato fruit. RIN, a MADS-box family transcription factor, and Nor, a NAC family transcription factor, are TFs that have been shown to be important in regulating ethylene biosynthesis and fruit ripening^26–28^. We found that the expression levels of *RIN* and *Nor* increased significantly at the beginning of fruit ripening in WT tomato, but significantly higher gene expression was found in *lcd1*. These data implicate the reduction in H_2_S levels in *lcd* in accelerated fruit ripening mediated by enhanced ethylene biosynthesis and the expression of ripening-related transcription factors.

Accumulating evidence suggests that exogenous H_2_S could inhibit the ripening and senescence of multiple fruits and vegetables by antagonizing the effects of ethylene and by regulating ROS metabolism^2,7–9^. In the present work, tomato fruit were sampled for transcriptome analysis at 0, 1, and 3 days post storage, and the expression of ripening-related genes was analyzed. Genes involved in cysteine and methionine metabolism pathways, selenocompound metabolism, porphyrin and chlorophyll metabolism, and plant hormone signal transduction were enriched in profile 17, and their expression increased in control fruit on day 3 compared to the expression in the H_2_S treatment group. We infer from our transcriptomic analysis that many genes in the methionine biosynthetic pathway, ethylene biosynthesis, ethylene signaling transduction, chlorophyll degradation, and fruit softening are significantly repressed by H_2_S.

Overall, we provide evidence that LCD1 in tomato is involved in sulfur metabolism in the nucleus and show that a reduction in endogenous H_2_S causes accelerated fruit ripening. However, we recognize that the role of endogenous H_2_S in plant development may be mediated at multiple levels, including by modulation of gene expression, by persulfidation, and by disturbed hormone generation due to changes in sulfur metabolism.

## Materials and methods

### Potential NLS analysis of LCDs in plants and subcellular localization analysis for SlLCD1 and SlLCD2 in tobacco cells

Putative l-cysteine desulfhydrase proteins were identified by the BLASTP tool in the Phytozome v12.1.6 (https://phytozome.jgi.doe.gov/pz/portal.html#) database and Sweetpotato Genomics Resource (http://sweetpotato.plantbiology.msu.edu/index.shtml) with the l-cysteine desulfhydrase AT3G62130 peptide sequence as a query. The plant species included *Arabidopsis thaliana*, *Brassica oleracea*, *Citrus sinensis*, *Cucumis sativus*, *Fragaria vesca*, *Glycine max*, *Ipomoea trifida*, *Ipomoea triloba*, *Malus domestica*, *Medicago truncatula*, *Oryza sativa*, *Populus trichocarpa*, *Solanum lycopersicum*, *Solanum tuberosum*, *Sorghum bicolor*, *Vitis vinifera*, and *Zea mays*. The potential NLS sites were obtained from NLS mapper^29^ (http://nls-mapper.iab.keio.ac.jp/cgi-bin/NLS_Mapper_form.cgi) with a criterion score of 5. The protein sequence of SlLCD1 (accession number: LOC101258894) was analyzed and showed a potential NLS at its N-terminus (H_18_LAKKPKLS). To explore the subcellular localization of the tomato LCD1 and LCD2 (accession number: XM_004238754.4) proteins, the full-length coding sequence for *SlLCD1* and *SlLCD2* was cloned into p1300-35S-GFP, and a series of SlLCD1 variants fused to GFP were generated: SlLCD1 (Δ1–26)-GFP, SlLCD1 (K21Q K22Q K24Q)-GFP, SlLCD1 (K21Q)-GFP, SlLCD1 (K22Q)-GFP, SlLCD1 (K24Q)-GFP, and SlLCD1 (K21Q K22Q)-GFP. Then, the generated fusion vectors were transformed into *Agrobacterium* strain GV3101. After overnight culture, the *Agrobacterium* cells were centrifuged and resuspended in 10 mM MgCl_2_, 10 mM 2-N-morpholino ethanesulfonic acid (MES), and 200 μM acetosyringone (AS) (pH 5.8) at an OD_600_ of 0.1–0.2. The mixtures were incubated at room temperature for 2 h and infiltrated into 4-week-old tobacco leaves. After infiltration, the plants were cultivated in the dark for 24 h at 16 °C and then cultivated for 48–72 h under 24 °C/16-h day and 18 °C/8-h night conditions. Hoechst 33342 was used to stain the nucleus^30^, while the chloroplasts were indicated by their autofluorescence. Cubic tobacco leaves were incubated with Hoechst 33342 (5 μg/mL) for 5 min. The excitation/emission wavelengths were 488 nm/507 nm for GFP, 346 nm/460 nm for Hoechst 33342, and 488 nm/650 nm to 750 nm for chlorophyll autofluorescence. All of the fluorescence signals were detected using a Zeiss LSM710NLO confocal laser scanning microscope. The primers used for the GFP vector are listed in Table S2.

### Enzymatic activity determination of SlLCD1 and SlLCD2 expressed in *E. coli*

The coding sequences of SlLCD1 and SlLCD2 were cloned into the expression vector pJC40 between the *Nde*I and *BamH*I restriction sites^31^. The recombinant plasmids were transformed into DE3 for protein expression. The transformant harboring pJC40-LCD1/2 (DE3) was grown in LB medium containing ampicillin at 37 °C overnight. The culture was then transferred to 100 mL of fresh LB medium containing ampicillin at 37 °C until the OD_600_ reached approximately 0.6–0.8. Isopropyl-β-D-thiogalactopyranoside (IPTG) was added at a final concentration of 1 mM and incubated overnight at 16 °C and 120 r.p.m. The bacterial cells were harvested by centrifugation at 12,000×*g* at 4 °C for 10 min and washed twice with 1 M phosphate buffer (pH 7.5 containing 1 mM PMSF) before resuspension. After ultrasound-mediated homogenization, the homogenate was centrifuged at 12,000×*g* and 4 °C for 20 min and analyzed to determine its ability to catalyze the decomposition of l-cysteine to release H_2_S according to the method described by Liu et al.^20^. The experiments were repeated three times, and the results are expressed as the mean ± SD (standard deviation). The related primers are listed in Table S3.

### VIGS of *SlLCD1* in tomato fruit

The TRV-based vectors pTRV1 and pTRV2 were used for VIGS of *SlLCD1* (accession number: LOC101258894). A 387-bp fragment corresponding to nt 29–416 of the *SlLCD1* sequence amplified from tomato cDNA by PCR was cloned into pTRV2 to generate pTRV2-SlLCD1. The primers used for pTRV2-SlLCD1 are listed in Table S3. *A. tumefaciens* strain GV3101 containing the TRV-VIGS vectors was injected on the surface of the fruit petiole as previously described^32^. *A. tumefaciens* strain GV3101 containing the vectors was cultured at 28 °C for 16 h in Luria-Bertani medium containing 20 μM AS, 10 mM MES, and 50 μg/mL each of the antibiotics kanamycin, gentamycin, and rifampicin. The *Agrobacterium* cells were harvested and resuspended in infiltration buffer containing 10 mM MgCl_2_, 10 mM MES (pH 5.6), and 200 μM AS and finally adjusted to an OD_600_ of 1.5. Resuspended cells with the pTRV1 and pTRV2 or pTRV2-SlLCD1 vector were then mixed together at a ratio of 1:1 and infected into pedicels of Micro-Tom tomato plants. Tomato petioles infiltrated with pTRV2 without the insert were used as controls. Tomato plants were then stored at 16 °C for 24 h and transferred to normal culture conditions thereafter. The changes in tomato fruit color were measured with a color difference meter (model WSC-100; Konica Minolta, Tokyo, Japan). L* indicates lightness, a* indicates chromaticity on a green (−) to red (+) axis, and b* stands for chromaticity on a blue (−) to yellow (+) axis. Each fruit was measured at four equidistant points around the middle area.

Tomato seeds were removed, and the flesh was sampled in VIGS-SlLCD1 fruit or *SlLCD1*-gene edited mutants. For tissue expression analysis of *SlLCDs*, different tissues of tomato were sampled. Total RNA from 0.1 g of frozen samples was extracted using the RNA Extraction Kit (Tiangen, Beijing, China), and cDNA was obtained using a reverse-transcription kit (PrimeScript RT Master Mix; Takara, Kyoto, Japan). The cDNA products were used for gene expression analysis by quantitative polymerase chain reaction (qPCR) performed using a Bio-Rad IQ5 (Hercules, CA). The specific primers used for qPCR were designed based on the coding sequence of the genes as shown in the SGN database (https://solgenomics.net/) or the NCBI database (https://www.ncbi.nlm.nih.gov/ncbisearch) (Table S4). Tubulin gene expression in control tomato plants was used for normalization of data. All analyses were repeated in three technical replicates. The heatmap of gene expression data was prepared by http://www.omicshare.com/tools.

### Determination of H_2_S content in tomato

For the H_2_S content assay, tomato fruit with VIGS-SlLCD1 or leaves from *SlLCD1*-gene edited mutants were sampled to analyze the H_2_S content as described^33^. The plant samples were homogenized with 1 mL of phosphate buffer solution (pH 7.0, 50 mM) containing 0.1 M ethylene diamine tetraacetic acid (EDTA) and 0.2 M ascorbic acid. Then, the homogenate was mixed with 1 mL of 1 M HCl and placed in a closed vial to release H_2_S. The released H_2_S was absorbed by a 1% (w/v) Zn(AC)_2_ (0.5 mL) trap that was placed in the vial. After incubation for 30 min, 100 μL of 20 mM *N*,*N*-dimethyl-p-phenylenediamine and 100 μL of 30 mM FeCl_3_ were added to the Zn(AC)_2_ solution. After 15 min in darkness, the amount of H_2_S released was determined by measuring the absorbance at 670 nm.

### CRISPR/Cas9 constructs for *SlLCD1* and transformation of *S. lycopersicum* cv. Micro-Tom

Two *SlLCD1* target sites (sgRNA1 and sgRNA2) were designed and selected by the CRISPR-P program (http://cbi.hzau.edu.cn/cgi-bin/CRISPR). The 20-bp oligos of sgRNA were integrated into the AtU3d and AtU3b vectors, and the sgRNA was assembled into the CRISPR/Cas9 binary plasmid by Golden Gate ligation^34^. *A. tumefaciens* containing the Cas9-SlLCD1 plasmid was used for stable transformation of tomato^35^. For confirmation of the *SlLCD1* gene-edited plants, genomic DNA was extracted, and the fragment flanking the sgRNA target sequence was amplified from genomic DNA and sent for sequencing. The sequences were decoded on the website http://skl.scau.edu.cn/dsdecode/^36^. The primer pairs used for vector construction and mutation analyses are listed in Table S4.

### Determination of the levels of chlorophyll, carotenoid, and cysteine in tomato fruit

The levels of chlorophyll and carotenoids in tomato fruit were determined according to the methods of Lichtenthaler and Wellburn^37^ and Nath et al.^38^, respectively. Tomato flesh (2.0 ± 0.1 g) was sampled from *lcd1* mutant fruit at 30, 35, 37, 40, and 42 DPA. Each analysis was repeated three times, and the chlorophyll and carotenoid levels were expressed as mg/g fresh weight (FW).

A cysteine assay kit (Beijing Solarbio Science & Technology Co., Ltd., China) was used to determine the cysteine content. Tomato flesh (0.2 g) as sampled from *lcd1* mutant fruit at 30, 35, 37, 40, and 42 DPA and assayed according to the instructions, and the absorbance was measured at 600 nm. The cysteine content was expressed as μmol/mL.

### Plant materials and transcriptome analysis

Tomato fruit at the white mature stage were fumigated with H_2_S released from 150 mL of 0.6 mmol/L sodium hydrosulfide (NaHS) solution or an equal amount of water. The tomato fruit and solutions were stored in 3-L containers at 23 ± 0.5 °C with a relative humidity of 85–90%. For transcriptomic analysis, tomato fruit were harvested at 0, 1, and 3 days post storage. Total RNA was extracted using the Plant RNeasy Extraction Kit (Qiagen, Germany). Sequencing of two biological replicates for each sample was performed using an Illumina GAII platform according to the manufacturer’s instructions. Different gene expression patterns were analyzed using the OmicShare tools, a free online platform for data analysis (http://www.omicshare.com/tools). The original gene expression data in FPKM (fragments per kilobase million) in CK 0d, CK 1d, H_2_S 1d, CK 3d, H_2_S 3d were compared with CK 0d and then calculated as log2 base values, and the data are shown in Supplementary data 1. The genes in profile 17 (shown in Supplementary data 2) were enriched by the KEGG database, and the related pathways were also exported by the OmicShare tools. The expression of ripening-related marker genes was illustrated in a heatmap by R (v3.3.1).

### Statistical analysis

Data were based on three replicates in each experiment, and the experiments were repeated independently three times. Statistical significance was assayed using a one-way analysis of variance with IBM SPSS Statistics (SPSS version 20.0; Armonk, NY), and the results are expressed as the means ± SDs. Significant differences were calculated by a *t*-test (*P* < 0.01 or *P* < 0.05) for significance.

## Supplementary information

Supplementary figures

supplementary tables

Supplementary data 1 The original gene expression data in FPKM (Fragments PerKilobase Million) in tomato of CK 0d, CK 1d, H_2_S 1d, CK 3d, H_2_S 3d were compared with CK 0d and then calculated in a log2 base

Supplementary data 2 The genes in profile 17 in Fig. 7B

Supplementary data 3 The transcription data of the genes in Fig. 7D

Supplementary data 4 The genes in profile 14 and 15 in Fig. 7B

Supplementary data 5 The differentially expressed genes in H_2_S treated fruit compared with control
