# Supplementary material for: A nuclear-localized cysteine desulfhydrase plays a role in fruit ripening in tomato
Source: Hortic Res. 2020 Dec 14;7:211. doi: 10.1038/s41438-020-00439-1 (PMC7736880; doi:10.1038/s41438-020-00439-1)
Supplement: Supplementary file 2 — supplementary tables [file 41438_2020_439_MOESM2_ESM.docx]

**Table S1.** The potential predicted nuclear localization signals (NLSs) in putative LCDs in various plant species.

| Gene name | Sequence ID | Predicted NLSs (The number following NLS stands for the position of first amino acid in NLS) |
| --- | --- | --- |
| *AtLCD1* | AT3G62130 | HRAPKKPRLA(16) |
| *AtLCD2* | AT5G26600 |  |
| *BolLCD* | Bol022276 | RRSKRYSSSTTNGLAAESVPDKRHKIS(19) |
| *CsaLCD* | Cucsa.090360 | HLPKRPKIAPTF(25)/CEKNHLPPDSTPHLNGDANTHLPKRPKIAPTF(5) |
| *CsiLCD1* | orange1.1g012971m.g | HHVSKKPKLT(12)/KKDGKMIRLAIIDHITSMPCVVIPVRKLVKI(185) |
| *CsiLCD2* | orange1.1g012921m.g | DTHLPKKPKLS(22)/PKKPKLSPTLISDTELESEFSHHVQNIARI(26) |
| *FvsLCD1* | gene22917-v1.0-hybrid | PASKKPKLAF(14) |
| *FvsLCD2* | gene22918-v1.0-hybrid |  |
| *GmaLCD1* | Glyma.20G092400 | PHPSKKIKLF(10)/GIRKRNHDLVV(329) |
| *GmaLCD2* | Glyma.03G228300 | GSHIPKKPKLSSTF(14) |
| *GmaLCD3* | Glyma.19G225600 | GSHIPKKPKLS(22) |
| *GmaLCD4* | Glyma.02G007800 |  |
| *GmaLCD5* | Glyma.10G137700 | RNGTAKKPKLT(21) |
| *itbLCD1* | itb10g13770.t2 |  |
| *itbLCD2* | itb07g21480.t1 | DNHLFKKQKLSF(55)/DNHLFKKQKLSF(365)  /IAAQKRWQLRFLQQPDDFFFNHLQKRILHS(95) |
| *itfLCD1* | itf10g13400.t1 |  |
| *itfLCD2* | itf07g19240.t1 | DNHLFKKQKLSF(23)/GIRKRNHDAVV(333)  /IAAQKRWQLRFLQQPDDFFFNHLQKRILHS(67) |
| *MdLCD1* | MDP0000177850 |  |
| *MdLCD2* | MDP0000305007 |  |
| *MdLCD3* | MDP0000267275 |  |
| *MtrLCD1* | Medtr1g484920 |  |
| *MtrLCD2* | Medtr7g111540 | HPTPKKPKLSSSSNF(19) |
| *MtrLCD3* | Medtr1g086070 | VQPDSKKPKLT(12)/WQLRFLQQPDDFFFNTLRNGILDSRKIIKN(63) |
| *OsaLCD1* | LOC_Os01g18640 | RPPPAKRPRSV(36) |
| *OsaLCD2* | LOC_Os01g18660 | PPSAKRPRAG(25)/NLHKWFFCPSAVAFIHTRKDDPVSSKLHHP(267) |
| *PtrLCD1* | Potri.005G003000 | NPTKKPRLSF(26)/SKHCKKNDSFNGCSNPTKKPRLS(12) |
| *PtrLCD2* | Potri.013G002400 | TPAKRTKLSF(19)/SLKHHKSHSLNGFTTPAKRTKLS(5) |
| *PtrLCD3* | Potri.002G187200 | QVSKKPRVSF(21) |
| *PtrLCD4* | Potri.014G112700 | QVSKKPRTS(17) |
| *SbiLCD1* | Sobic.003G138900 | GPSPKRARSV(34) |
| *SbiLCD2* | Sobic.003G139000 |  |
| *SitLCD1* | Seita.5G032200 | GPSPAKRPRAV(30) |
| *SitLCD2* | Seita.5G032100 |  |
| *SlLCD1* | LOC101258894 | HLAKKPKLS(18)/IAAQKRWQLRFLQQPDDFFLNHLQKRILHS(65) |
| *SlLCD2* | XM_004238754.4 | RAGGKVIEVHLPFPLNSNEEIVTEFDKALKM(148) |
| *StuLCD1* | PGSC0003DMG400031255 | RAGGKVIEVHLPFPLNSNEEIITEFDKALKM(148) |
| *StuLCD2* | PGSC0003DMG400001121 | HLAKKPKLS(18) |
| *VviLCD1* | GSVIVG01031326001 |  |
| *VviLCD2* | GSVIVG01028252001 | HVSKKPKLSAF(43) |
| *ZmaCEF1* | GRMZM2G322186 | GPSPSKRPRAV(26) |
| *ZmaLCD1* | GRMZM2G077212 |  |

The plant species include *Arabidopsis thaliana*, *Brassica oleracea*, *Citrus sinensis*, *Cucumis sativus*, *Fragaria vesca*, *Glycine max*, *Ipomoea trifida*, *Ipomoea triloba*, *Malus domestica*, *Medicago truncatula*, *Oryza sativa*, *Populus trichocarpa*, *Solanum lycopersicum*, *Solanum tuberosum*, *Sorghum bicolor*, *Vitis vinifera* and *Zea mays*.

**Table S2.** Primers used for protein subcellular localization of SlLCD1 and SlLCD2.

| **Primers** | **Sequence (from 5’ to 3’)** |  |
| --- | --- | --- |
| SlLCD1-GFP-F | 5’GAGAACACGGGGGACTCTAGAATGGAACCGGCGAACG3’ |  |
| SlLCD1-GFP-R | 5’GCCCTTGCTCACCATGGATCCTTCTGAGTGAAGCATCTTAC3’ |  |
| SlLCD1-∆1-26-F | 5’GAGAACACGGGGGACTCTAGAATGACCAGATCGTCTTCTCTCATTACG3’ |  |
| SlLCD1-K21Q/K22Q/K24Q-GFP-F | 5’GAGAACACGGGGGACTCTAGAATGGAACCGGCGAACGACGATGACCACCGAGCTAATGGCTCCGACCACAGTCATTTGGCTCAGCAACCTCAGCTGTCCAC3’ |  |
| SlLCD1-K21Q-F | 5’AGAACACGGGGGACTCTAGAATGGAACCGGCGAACGACGATGACCACCGAGCTAATGGCTCCGACCACAGTCATTTGGCTCAGA3’ |  |
| SlLCD1-K22Q-F | 5’GAGAACACGGGGGACTCTAGAATGGAACCGGCGAACGACGATGACCACCGAGCTAATGGCTCCGACCACAGTCATTTGGCTAAGCAAC3’ |  |
| SlLCD1-K24Q-F | 5’CACGGGGGACTCTAGAATGGAACCGGCGAACGACGATGACCACCGAGCTAATGGCTCCGACCACAGTCATTTGGCTAAGAAACCTCAGC3’ |  |
| SlLCD1-K21Q/K22Q-F | 5’GAGAACACGGGGGACTCTAGAATGGAACCGGCGAACGACGATGACCACCGAGCTAATGGCTCCGACCACAGTCATTTGGCTCAGCAAC3’ |  |
| SlLCD2-GFP-F | 5’GAGAACACGGGGGACTCTAGACATGTCTTCCGGTGACTTC3’ |  |
| SlLCD2-GFP-R | 5’GCCCTTGCTCACCATGGATCCATTCGAGAGAATTGCACAAG3’ |  |

The red bases stand for the positions induced the mutation from lysine (K) to glutamine (Q).

**Table S3**. Primers used for constructing pTRV2-SlLCD1 and pJC40-SlLCD1/2 construction.

| Primers | Sequences (5’-3’) |
| --- | --- |
| TRV2-LCD1-F | 5’GTGAGTAAGGTTACCGAATTCAGCTAATGGCTCCGACCACA3’ |
| TRV2-LCD1-R | 5’CGTGAGCTCGGTACCGGATCCCCTTTCTTAAACCGTCCCTCCG3’ |
| SlLCD1-EXP-F | 5’CATATCGAAGGTCGTCATATGATGGAACCGGCGAACGACG3’ |
| SlLCD1-EXP-R | 5’CTAGGGCCCGGGATCCTCGAGTTATTCTGAGTGAAGCATCTTACAA3’ |
| SlLCD2-EXP-F | 5’CATATCGAAGGTCGTCATATGATGTCTTCCGGTGACTTCCG3’ |
| SlLCD2-EXP-R | 5’CTAGGGCCCGGGATCCTCGAGTCAATTCGAGAGAATTGCACAAG3’ |

**Table S4**. Primers for constructing CRISPR/Cas9-SlLCD1 and primers for amplification of a fragment containing the target site of gRNA of CRISPR/Cas9-SlLCD1 in transformed tomato seedlings.

| Gene | Primer names | Forward primer (5’-3’) | Reverse primer (5’-3’) |
| --- | --- | --- | --- |
| Primers for constructing CRISPR/Cas9-SlLCD1 | | | |
| *SlLCD1* | F1/R1 | GTCAGTTCGCCCATCATCAGACC | AAACGGTCTGATGATGGGCGAAC |
|  | F2/R2 | ATTGAAGCTTATGTCACCCGGGC | AAACGCCCGGGTGACATAAGCTT |
| Primers for amplification of a fragment containing the target site of gRNA of CRISPR/Cas9-SlLCD1 | | | |
| *SlLCD1* | F/R | TAATGGCTCCGACCACAGT | CACGCACAGGTATGACAACAC |

**Table S5.** Primers used for quantitative RT-PCR.

| Primers | Sequence | Gene ID |
| --- | --- | --- |
| *SlLCD1* (forward) | GATGGCTACGTGACAGGATATG | LOC101258894 |
| *SlLCD1* (reverse) | CTCGCACAAGCTGAAGAATTG |  |
| *SlLCD2* (forward) | AGATCGGAGAAGGTGGTAGAT | NC_015442 |
| *SlLCD2* (reverse) | CACTATAATCCCTCGTCCCAATC |  |
| *SlSGR1* (forward) | GGCTATCTCCCAAACCATCAA | Solyc08g080090 |
| *SlSGR1* (reverse) | ACTCTGCAACAACTTCATCTCT |  |
| *SlPPH* (forward) | CAGATGTCGTGGATGGGAAA | Solyc01g088090 |
| *SlPPH* (reverse) | GCCTTTAGCTTCACTTGGTAAAC |  |
| *SlPAO* (forward) | CCTCATCGTCTTGCTCCTTTAT | Solyc11g066440 |
| *SlPAO* (reverse) | GCAGCTTGAGGTATCCTTGT |  |
| *SlNYC1* (forward) | GATCAGGGAAGAGCACTGTATG | Solyc07g024000 |
| *SlNYC1* (reverse) | GAGAACACAGACACCCAAGTAT |  |
| *SlPSY1* (forward) | GAAGATGCCAGAAGAGGAAGAG | Solyc03g031860 |
| *SlPSY1* (reverse) | GGTCACCCTTCCAGCAAATA |  |
| *SlPDS* (forward) | CAAGACCAGAGCTGGACAATAC | Solyc03g123760 |
| *SlPDS*(reverse) | CAAACCTGCACCAGCAATAAC |  |
| *SlZDS* (forward) | GATTGGTTCCTCAGAAGTGGAG | Solyc01g097810 |
| *SlZDS* (reverse) | CCAGCCATTGTAGCGTAGTT |  |
| *SlE4* (forward) | CATGACCCGACCACTCTAAATC | Solyc03g111720 |
| *SlE4* (reverse) | CAGTTGAGCCTGAGCATCAT |  |
| *SlE8* (forward) | GTCTCGGTCTCGATCGTAGTTA | Solyc09g089580 |
| *SlE8* (reverse) | AGGACATGGTGGGTAGTAGTT |  |
| *SlACO1* (forward) | CCATGTCCTAAGCCCGATTT | NM_001247095.2 |
| *SlACO1* (reverse) | GGCCACTCACTTTGTCATCT |  |
| *SlACO3* (forward) | GCGCCATCTTCCTACTTCTAAT | Solyc07g049550 |
| *SlACO3* (reverse) | CTCAGCCAACTTCTCCAATCT |  |
| *SlACS2* (forward) | GAGGTTCGTAGGTGTTGAGAAA | Solyc01g095080 |
| *SlACS2* (reverse) | GGAGGAATAGGTGACGAAAGTG |  |
| *SlPG* (forward) | TTGGAGGAGGAGGAACTATCA | Solyc10g080210 |
| *SlPG* (reverse) | CCTGCATGGCAGTGATTTATTT |  |
| *SlCEL2* (forward) | CTGCTGTTTGCCCTTTCTATTG | Solyc09g010210 |
| *SlCEL2* (reverse) | TGCCTTCTTCTTGTTGCTTTATG |  |
| *SlEXP* (forward) | CCGACGATTGGACACCTAAA | Solyc06g051800 |
| *SlEXP* (reverse) | GGTCCTCCTTAATCAAAGGACATA |  |
| *SlTBG4* (forward) | GCCATTGGCCTGGATACATA | Solyc12g008840 |
| *SlTBG4* (reverse) | AGAAGGTTGTCCGCAGTTAG |  |
| *SlXTH5* (forward) | AGGATTCAGCCATCTCTTTGG | Solyc01g081060 |
| *SlXTH5* (reverse) | ACTTGAACCCTGAACCTGTG |  |
| *SlRIN* (forward) | CAGCTTGAACGTCAATTGGATTCAT | NM_001247741.2 |
| *SlRIN* (reverse) | CTTTGCTCACCACAATGCCATGA |  |
| *SlNOR* (forward) | TCCATGGGAACTCCCTGCTAAGG | NM_001247723.2 |
| *SlNOR* (reverse) | CCTTTTGTGTTCCACCGGAAGTAAA |  |
| *SlTubulin* (forward) | TAGAGCCTGGTACGATGGATAG | Solyc08g006890 |
| *SlTubulin* (reverse) | CAACTCAGCGCCTTCAGTATAA |  |
