# Supplementary material for: A nuclear-localized cysteine desulfhydrase plays a role in fruit ripening in tomato
Source: Hortic Res. 2020 Dec 14;7:211. doi: 10.1038/s41438-020-00439-1 (PMC7736880; doi:10.1038/s41438-020-00439-1)
Supplement: Supplementary file 1 — Supplementary figures [file 41438_2020_439_MOESM1_ESM.docx]

**Fig. S1** The gene expression of *SlLCD1* and *SlLCD2* in different parts or different development stages of tomato plant in the cultivar of tomato Micro-Tom.

**Fig. S2** The intended mutations of SlLCD1 on potential nuclear localization signal. A. The K (Lysine) residues in SlLCD1 were substituted with Q (Glutamine). B. The schematic diagram of intended mutations on SlLCD1.

**Fig. S3** Enzymatic activity assay of SlLCDs in *E. coli*. A. The *SlLCD1* and *SlLCD2* coding sequences were cloned into pJC40 and expressed and induced by IPTG in BL21 cells. The empty pJC40 was used as a negative control. The boxes indicated the expressed proteins of SlLCD1 and SlLCD2. B. The enzyme activities of the recombinant proteins were determined in the present of L-cysteine and both SlLCD1 and SlLCD2 showed strong ability to produce H_2_S. Values are mean of three replicated ±SD in the basis of protein content. ** stands for P < 0.01 after Student’s *t*-test.

**Fig. S4** Plant phenotype of tomato *SlLCD1* homozygous mutants *lcd1-7* and *lcd1-9*. (A) Image of the *lcd1-7* and *lcd1-9* mutant plants in comparison to wild type. (B) Plant height of the wild type and mutants during 100 days of growth. Values are means ±SD of three replicates. The symbol ** stands for P<0.01.

**Fig. S5** Relative gene expression of *NYC1*, *PAO*, *PPH*, *SGR1*, *PDS*, *PSY1* and *ZDS* in fruits of wild type and *lcd1-7* mutant plants during 40 days post anthesis (DPA). Values are means ±SD of three replicates. The symbol ** stands for P<0.01.

**Fig. S6** Relative gene expression of *CEL2*, *EXP*, *PG*, *TBG4* and *XTH5* in fruits of wild type and *lcd1-7* mutant plants during 40 days post anthesis (DPA). Values are means ±SD of three replicates. The symbol ** stands for P<0.01.

**Fig. S7** Relative gene expression of *ACO1*, *ACO3*, *ACS2*, *E4*, *E8*, *RIN* and *Nor* in fruits of wild type and *lcd1-7* mutant plants during 40 days post anthesis (DPA). Values are means ±SD of three replicates. The symbol ** stands for P<0.01.

**Fig. S8** Differentially expressed genes in tomato fruits treated with H_2_S or in control for 0, 1 and 3 days and the KEGG pathway analysis based on genes enriched in profile 17 as shown in Fig. 1b.

**Fig. S8A** Differentially expressed genes in tomato fruits treated with H_2_S or in control (CK) for 1 and 3 days.

**Fig. S8B** The cysteine and methionine metabolism pathway.

**Fig. S8C** The selenocompound metabolism.

**Fig. S8D** Porphyrin and chlorophyll metabolism.

**Fig. S8E** Plant hormone signal transduction.

**Fig. S9** KEGG (A) and GO (B) analysis based on the genes in Profile 14 and 15 as shown in Fig. 7B.

**Fig. S10** KEGG analysis of up-regulated genes in tomato fruit treated with H_2_S for 1 day compared with control.

**Fig. S11** KEGG analysis of down-regulated genes in tomato fruit treated with H_2_S for 1 day compared with control.

**Fig. S12** KEGG analysis of up-regulated genes in tomato fruit treated with H_2_S for 3 day compared with control.

**Fig. S13** KEGG analysis of down-regulated genes in tomato fruit treated with H_2_S for 3 day compared with control.
